# Hypoxia-Inducible Factor-1 as a Therapeutic Target in Endometrial Cancer Management

**DOI:** 10.1155/2010/580971

**Published:** 2010-02-14

**Authors:** Laura M. S. Seeber, Ronald P. Zweemer, René H. M. Verheijen, Paul J. van Diest

**Affiliations:** ^1^Department of Gynaecological Oncology, University Medical Centre Utrecht, PO Box 85500, 3508 GA Utrecht, The Netherlands; ^2^Department of Pathology, University Medical Centre Utrecht, PO Box 85500, 3508 GA Utrecht, The Netherlands

## Abstract

In the Western world, endometrial cancer (EC) is the most common malignant tumor of the female genital tract. Solid tumors like EC outgrow their vasculature resulting in hypoxia. Tumor hypoxia is important because it renders an aggressive phenotype and leads to radio- and chemo-therapy resistance. Hypoxia-inducible factor-1*α* (HIF-1*α*) plays an essential role in the adaptive cellular response to hypoxia and is associated with poor clinical outcome in EC. Therefore, HIF-1 could be an attractive therapeutic target. Selective HIF-1 inhibitors have not been identified. A number of nonselective inhibitors which target signaling pathways upstream or downstream HIF-1 are known to decrease HIF-1*α* protein levels. In clinical trials for the treatment of advanced and/or recurrent EC are the topoisomerase I inhibitor Topotecan, mTOR-inhibitor Rapamycin, and angiogenesis inhibitor Bevacizumab. Preliminary data shows encouraging results for these agents. Further work is needed to identify selective HIF-1 inhibitors and to translate these into clinical trials.

## 1. Introduction

Endometrial cancer is the most common malignant tumour of the female genital tract. The American Cancer Society estimates that 42.160 women will have been diagnosed with, and 7780 women will have died of cancer of endometrial cancer in 2009 in the US [1]. Ninety percent of endometrial cancer cases are sporadic, while the remaining are deemed hereditary. In the endometrium, different adenocarcinoma subtypes can develop. Endometrioid endometrial carcinoma (EEC), or Type 1 cancer, accounts for approximately 75% of cases. These tumours are usually oestrogen dependent, tend to be of lower grade, and have fewer recurrences and a better survival. They often develop in a background of adenomatous hyperplasia and are characterized by mutations in PTEN and defects in DNA mismatch repair—as manifested by microsatellite instability. Type 2 tumours, of which serous endometrial carcinoma (USPC) is the most common subtype, arise from atrophic endometrium. Type 2 tumours often show p53 and are usually nondiploid. USPCs are often poorly differentiated and have a greater propensity for early spreading. They have worse prognosis than that of EEC. 

**Figure 1:**
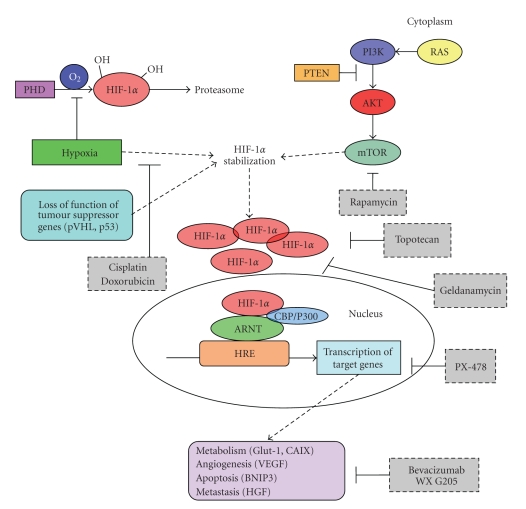
Mechanisms of HIF activation in cancer.

A developing solid tumour will outgrow its own vasculature beyond the size of several cubic millimetres, resulting in hypoxia (defined as an oxygen tension below the physiological level, <2% pO_2_) [2]. Hypoxia has been found to be an important event in carcinogenesis as it renders a more aggressive phenotype with increased invasiveness and proliferation, formation of metastases, and poorer survival in several types of cancer [3, 4]. Furthermore, it has been shown that hypoxia-induces resistance to radiotherapy and chemotherapy [5–7]. The key survival gene for cells in a hypoxic environment is hypoxia inducible factor-1 (HIF-1) (see Figure 1). 

The unsatisfactory results obtained with conventional pharmacological treatment encourage further biological and clinical investigations addressed to a better understanding of specific cell targets and signalling transduction pathways involved in endometrial carcinogenesis and to the identification of novel molecular therapeutic targets. As hypoxia, and thus HIF-1, leads to resistance to radiotherapy and chemotherapy in solid tumours [5–8], targeting HIF-1 could be an attractive treatment strategy, with the potential for disrupting multiple pathways crucial for tumour growth. In this review, we will describe the current status of HIF-1 (upstream and downstream) inhibitors in the treatment of endometrial cancer.

## 2. Hypoxia-Inducible Factor-1*α*

HIF-1 is a transcription factor composed of the subunits HIF-1*α* and HIF-1*β*, which are basic helix-loop-helix DNA binding proteins. Both HIF-1*α* and HIF-1*β* genes are ubiquitously expressed and heterodimerize to form the active HIF-1 that activates gene transcription by binding to the consensus HIF Responsive Element (HRE): 5′-RCGTG-3′ in promoters and enhancers of target genes [9]. The activity of HIF-1 is predominantly regulated at the post-translational level by regulating HIF-  1*α*  protein stability. At normal oxygen tension, HIF-1*α* is hydroxylated by prolyl hydroxylases (PHD) in the oxygen dependent degradation domain (ODDD). Hydroxylated HIF-1*α* is recognized by the Von Hippel-Lindau (VHL) protein, ubiquitinated and destined for degradation by proteasomes. This process is inhibited during hypoxia [10]. Under hypoxia, stabilized HIF-1*α* subunits heterodimerize with *β* subunits (also known as ARNT) to transactivate target genes after nuclear translocation. Among these are genes involved in adaptation to low glucose levels like the glucose transporter Glut-1, carboanhydrase IX (CAIX) that regulates pH [11], and vascular endothelial growth factor (VEGF) that is one of the most potent inducers of angiogenesis [12]. Although HIF-1*α* usually induces prosurvival (CAIX, Glut-1, VEGF) genes, a role of HIF-1*α* in regulation of apoptosis has also been described. HIF-1*α* promotes cell death through an increase in p53 or other proapoptotic proteins like BNIP3 [13]. As a result of this dual function of HIF-1*α*, a “stop-and-go” strategy as a dynamic balance to maintain overall cell growth and survival has been proposed [14]. Hypoxia induced HIF-1*α* also affects the expression of genes involved in metastasis formation. Hepatocyte growth factor (HGF), for example, is a cytokine which stimulates proliferation and invasion through its receptor, the protooncogene c-MET [15]. Invasive cell growth is promoted by HIF-1*α* -induced c-Met transcription and sensitizing of cells to HGF stimulation [16–18]. Taken together, the adaptive response to hypoxia in primary tumours resembles in many ways the so-called metastatic phenotype which explains the poor prognosis of hypoxic cancers [19]. 

HIF-1 stabilization may also occur under oxygen-independent conditions, including infection with oncogenic viruses, loss-of-function mutations in tumour suppressor genes such as Von Hippel-Lindau (VHL), or signaling by receptor tyrosine kinases, prostaglandin E2 receptor, or nitric oxide [20]. Furthermore, genetic alterations in the EGFR [21], RAS, and PI-3K/Akt [22–24] as well as loss of p53 function [25] have been shown to lead to increased nonhypoxic HIF activity. HIF-1*α* has also been shown to be regulated by mammalian target of Rapamycin (mTOR). mTOR promotes increased translation of HIF-1*α* mRNA into protein [26, 27]. Other possible mechanisms contributing to normoxic HIF-1 expression like oncogenic mutation or amplification of HIF-1*α* gene have rarely been reported in solid cancers [28, 29]. A polymorphism in HIF-1*α* (P582S) has been found associated with increased HIF activity and poor prognosis in prostate cancer, but its significance with cancer risk is still incompletely understood [30, 31]. The single nucleotide polymorphism (SNP) C1772T (also described as C1744T) in the HIF-1*α* gene coding region results in an amino acid change at position 582 changing a Proline to a Serine (i.e., P582>S) in the ODD domain (http://www.ncbi.nlm.nih.gov/SNP/snp_ref.cgi?rs=11549465). Carriers of this SNP seemed to have an increased risk of developing cervical and endometrial cancer [32]. However, the proportion of allele carriers with the most common polymorphism in the control group was different from ratios described in other studies. We [31] examined whether the C1744T polymorphism increased the risk for endometrioid endometrial cancer. Although the C1744T polymorphism was associated with higher microvessel density and AKT activation, it did not lead to increased cancer risk. Interestingly we found that the P582S genotype variation in the ODDD of the HIF-1*α* protein may occur as a de novo mutation in endometrial cancer. Although the significance of this remains to be established, others have proposed that it may increase transactivation of HIF-1*α* [30].

## 3. Endometrial Cancer

### 3.1. HIF-1*α* and Endometrial Carcinogenesis

It has been postulated that menstruation results from vasoconstriction of spiral arterioles, causing hypoxia which leads to necrosis [33]. This focal hypoxia in perimenstrual endometrium could result in locally increased HIF-1*α*. However, in premenopausal women, HIF-1*α* was undetectable in the majority of samples. In the HIF-1*α* positive cases, expression was only seen in a small focus within the tissue, suggesting that if hypoxia does occur at this time, then it is not widespread. There seemed to be no correlation of HIF-1*α* expression and the menstrual cycle [34]. In postmenopausal women, HIF-1*α* was increasingly overexpressed from inactive endometrium through hyperplasia to endometrioid carcinoma, paralleled by activation of its downstream genes such as CAIX, Glut-1, VEGF, and increased angiogenesis. Low HIF-1*α* expression was associated with negative/low VEGF staining in the total group [35]. These results highlight the potential importance of hypoxia and its key regulator HIF-1*α* in endometrial carcinogenesis and progression of disease [36].

Perinecrotic, chronic hypoxia-associated HIF-1*α* expression was absent in inactive endometrium, rare in endometrial hyperplasia, and frequent in endometrioid carcinoma. These results could point to the importance of hypoxia and the subsequent stabilization of HIF-1*α* in endometrial carcinogenesis [35–38]. Loss of PTEN tumour suppressor gene (also known as MMAC1) is often seen in endometrial carcinogenesis and is thought to cause nonhypoxia-mediated HIF-1*α* expression via activation of the PI3K/AKT and mTOR signaling pathway [39–42]. Horrée et al. (unpublished data) showed that although over 60% of the carcinomas showed extensive loss of PTEN by immunohistochemistry, this was not correlated to increased HIF-1*α* expression. Thus, diffuse nonhypoxia-related expression of HIF-1*α* seemed not to be related to PTEN mutation in endometrial cancer. Correlation of HIF-1*α* with tumour stage, tumour grade, or myometrial invasion is still under discussion [35, 43, 44]. 

The mechanism of tumourigenesis of USPC differs from that of EEC. More expression of HIF-1*α* was observed in USPC than in EEC [44, 45]. In USPC, HIF-1*α* expression was not correlated to clinical stage or depth of myometrial invasion. p53 mutations are a common event in USPC carcinogenesis and aberrant p53 accumulation has been associated with HIF-1*α* overexpression in different human tumours [46]. In contrast, p53 expression was not associated with HIF-1*α* expression in type II endometrial carcinomas [44, 45]. 

### 3.2. HIF-1*α* and Prognosis in Endometrial Cancer

**Figure 2:**
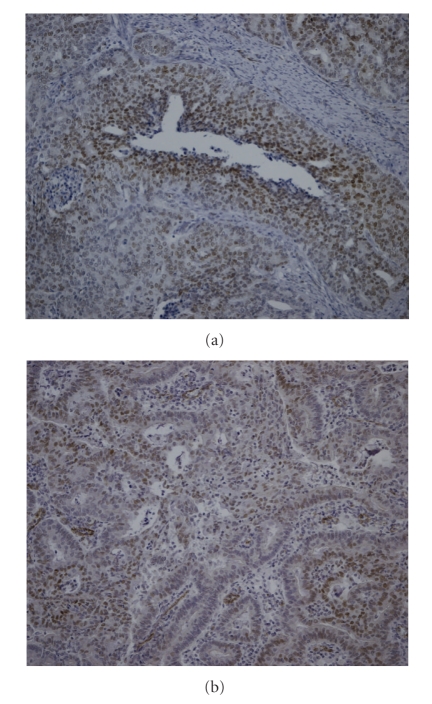
Immunohistochemical staining of HIF-1*α* in endometrioid endometrial carcinoma. Typical patterns are shown: (a) perinecrotic HIF-1*α* expression (10× magnification) and (b) diffuse HIF-1*α* expression (10× magnification).

Contradictory results have been described as to the prognostic value of HIF-1*α* overexpression in endometrial carcinoma. HIF-1*α* showed significantly higher expression in recurrent endometrial carcinomas when compared with their primary tumours; it was, however, not an independent predictor for recurrent endometrial carcinoma [43, 44]. In stage 1 endometrial cancers, HIF-1*α* was associated with a worse prognosis [37]. However, others did not find prognostic impact of HIF-1*α* expression [38]. Besides the limitation of relatively small numbers of patients in these studies, immunohistochemical studies are difficult to compare because of a variation in definition of HIF-1*α* positivity. In some studies, both nuclear and cytoplasmic staining was scored. The significance of cytoplasmic HIF-1*α*, however, is still not clear as stable HIF-1*α* is thought to rapidly translocate to the nucleus. Expression patterns that can be observed in endometrial tumours are the diffuse, perinecrotic, and mixed (both perinecrotic and diffuse) patterns [35]. Perinecrotic HIF-1*α* expression is thought to be hypoxia driven, whereas diffuse HIF-1*α* expression may rather be due to nonhypoxic stimuli [47]. Our experience shows that once authors take into account nuclear staining only and HIF-1*α* expression pattern, the results can change dramatically. Figure 2 shows an example of nuclear HIF-1*α* in a diffuse and perinecrotic expression pattern. 

As HIF-1*α* expression is associated with treatment failure and/or patient mortality, targeting HIF-1*α* could be an attractive treatment strategy, with the potential for disrupting multiple pathways crucial for tumour growth.

## 4. HIF-1*α* and Hypoxia as a Target for Cancer Therapy

The unsatisfactory results obtained with conventional pharmacological treatment encourage further biological and clinical investigations addressed to a better understanding of specific cell targets and signaling transduction pathways involved in endometrial carcinogenesis and to the identification of novel molecular targeted therapies. A new and more effective treatment for metastatic endometrial carcinoma is urgently needed.

There are different areas of research in hypoxia-related drug therapy including (1) designing drugs that directly inhibit HIF-1 signaling and (2) influencing other signaling cascades that indirectly alter HIF signaling. 

Inhibition of HIF-1*α* would, of course, hit multiple targets but because of its bifunctional effects, for example, proapoptotic genes induced by hypoxia, outcome will be difficult to predict. Thus far, selective HIF-1 inhibitors have not been identified. A number of nonselective inhibitors, which indirectly target signaling pathways upstream or downstream HIF-1 are known to decrease HIF-1*α* protein levels. Antisense therapy against HIF-1*α* has been shown to reduce HIF-1*α* expression and transcriptional activity; however, at present it is not clinically applicable. Therefore, the potential of HIF-1*α* as a target for cancer therapy lies in the small molecule inhibitors of HIF-1. Several small molecular inhibitors of the HIF-1 transcriptional activation pathway have been identified (Table 1). Although none of these has been shown to directly and specifically target HIF-1 [54, 55], they do decrease HIF-1*α* protein levels. Some of these HIF-1 inhibitors are subject of clinical trials at present.

### 4.1. Topotecan

Topotecan, a topoisomerase I inhibitor that has been used as a second-line therapy for ovarian cancer, is one such small molecule inhibitor of HIF-1 [56, 57]. Topotecan inhibits hypoxic induction of HIF-1*α* protein and DNA binding activity [58]. In a GOG phase II trial of Topotecan in pretreated patients with advanced, persistent, or recurrent endometrial carcinoma, the total response rate was 9%, with 1 patient achieving a complete response and 1 experiencing a partial response. Twelve (55%) patients maintained stable disease [48]. The eastern Cooperative Oncology Group subsequently performed a phase II trial of Topotecan for metastatic or recurrent endometrial carcinoma. The overall response was 20%. Although single-agent Topotecan treatment has shown activity in chemonaïve [50] and previously treated patients [48, 49], severe (grade 4 neutropenia) and unexpected (primarily sepsis) toxicities were encountered [48, 50]. However, at modified doses, toxicity was acceptable and clinical activity was preserved [50]. 

Despite the different carcinogenesis of EEC and USPC, but probably due to the rareness of the latter, clinical trials including only USPC patients are rare. A pilot study of Topotecan for the treatment of USPC demonstrated clinical activity in this patient group [59]. However, because survival outcomes continue to be disappointing, combining Topotecan with other active drugs may lead to improved outcomes.

### 4.2. mTOR Inhibitors

HIF-1*α* has also been shown to be regulated by mammalian target of Rapamycin (mTOR). mTOR promotes increased translation of HIF-1*α* mRNA into protein [26, 27]. Rapamycin is a specific inhibitor of mTOR and inhibits both the stabilization and the transcriptional activity of HIF-1*α* in hypoxic cancer cells [60]. This effect is directly related to the disruption of mTOR-dependent signaling functions. The current hypothesis is that mTOR inhibitors could be effective inhibitors of hypoxic adaptation in developing tumours. These effects could be especially relevant in tumours with loss of PTEN function as is often the case in endometrioid endometrial cancer [61]. Loss of PTEN leads to constitutive activation of AKT, which leads to up-regulation of mTOR. Potential therapies targeting the mTOR pathway include mTOR inhibitors (Rapamycin derivates) Temsirolimus (CCI-779), and Everolimus (RAD001) [62]. Demonstrated activity in preclinical studies has led to numerous phase I and phase II trials. A phase II trial of Temsirolimus in patients with chemotherapy treated, recurrent, or metastatic endometrial cancer showed modest activity of Temsirolimus. Two patients (7.4%) showed partial response and twelve patients (44%) had stable disease [51]. A 44% clinical benefit response rate was found in a phase II study of Everolimus in 29 patients with recurrent endometrial cancer [52]. Clinical Benefit was defined as complete or partial response or prolonged stable disease. In this trial, loss of PTEN expression was predicative of response rate. The different mTOR inhibitors show encouraging single agent clinical benefit. A phase I trial of Temsirolimus with Topotecan (NCT00523432) in patients with gynaecological malignancies, including endometrial cancer, has just finished recruiting patients. Other trials of Temsirolimus are underway.

### 4.3. Bevacizumab

An HIF-1 inhibitor that targets a pathway activated by HIF-1 is Bevacizumab. Bevacizumab is a monoclonal antibody that targets VEGF, a potent endothelial cell mitogen that has been associated with increased angiogenesis in malignancies. Different studies showed that an increase in VEGF expression was linked to increased angiogenesis in endometrial carcinomas. High VEGF mRNA levels were correlated significantly with highly vascularized tumours [63, 64]. Early results of a phase II study of Bevacizumab in the treatment of recurrent or persistent endometrial cancer in 53 patients showed a 15% response rate. Nearly 36% of the patients were progression free at 6 months [53]. In conclusion, Bevacizumab appears to be active in women with recurrent or persistent endometrial cancer. 

### 4.4. Cisplatin and Doxorubicin

Some conventional anticancer agents targeting signal transduction pathways have also been shown to inhibit HIF-1 [65]. Duyndam et al. [66] showed in human ovarian cancer cell lines that the conventional anticancer agents cisplatin and doxorubicin can negatively influence HIF-1 activity with a concomitant reduction of VEGF expression. A recent phase III trial demonstrated improved progression-free and overall survival for the three-drug regimen of cisplatin, doxorubicin and paclitaxel compared with a two-drug combination (cisplatin and doxorubicin) in advanced or recurrent endometrial carcinoma. However, toxicity problems often make the three-drug regimen less acceptable [67].

### 4.5. New Promising Drugs

Small molecule inhibitors of HIF-1 activity currently investigated in clinical trials are PX-478, an inhibitor of HIF-1 transcription factor activity [68], and geldanamycin, an HSP90 (heat shock protein 90) inhibitor [69]. HSP90 is involved in the folding of HIF-1*α* and Geldanamycin induces degradation of HIF-1*α* [70]. Both are being evaluated in advanced solid tumours. WX G250, a CAIX antibody (http://www.wilex.com), is another HIF-1 inhibitor that targets a different pathway activated by HIF-1. WX G250 is currently in phase III clinical trials in renal cell cancers. These new drugs may find their way into clinical trials in endometrial cancer in the future.

## 5. Summary

Hypoxic tumours are usually resistant to radiotherapy and conventional chemotherapy, rendering them highly aggressive and metastatic. Response to hypoxic stress is largely mediated by the HIF pathway. HIF-1*α* expression is correlated with a poor prognosis in endometrial cancer. Therefore, targeting the HIF pathway provides an attractive strategy to treat hypoxic and highly angiogenic tumours. Thus far, selective HIF-1 inhibitors have not been identified. A number of nonselective inhibitors, which indirectly target signaling pathways upstream or downstream of HIF-1, are known to decrease the key regulating HIF-1*α* protein levels. Different (indirect) HIF-1*α* inhibitors that are in clinical trial for the treatment of advanced/recurrent endometrial carcinoma are Topotecan, Rapamycin derivates, and Bevacizumab. Preliminary results show encouraging results for these single-agent treatments with partial response and stable disease in the patients. However, lack of specificity increases the difficulty in attributing any antitumorigenic effects of these drugs specifically to inhibition of HIF-1. The combination of HIF inhibitors with conventional treatment may prove to be clinically useful. Further work is needed to identify more selective HIF-1 inhibitors, to determine their mechanism of action, and to translate these developments into clinical trials.

## Figures and Tables

**Table 1:** Clinical trials on HIF-1*α* targeted therapies in endometrial cancer.

Class	Inhibitor	Mechanism	Clinical trials in endometrial cancer
*Small molecule inhibitors of HIF-1*			
Topoisomerase inhibitor	Topotecan (topo-I)	Inhibits hypoxic induction of HIF-1*α* protein and DNA binding activity	Miller et al. [48] Triana et al. [49] Wadler et al. [50]
HSP90 inhibitor	Geldanamycin	Induces degradation of HIF-1*α* protein and inhibition of DNA binding of HIF-1	—
Other	PX-478	Inhibition of HIF-1*α* transcription activity	—

*Inhibitors of signal transduction pathways*			
mTOR inhibitor	Temsirolimus (CCI-779)	Downregulation of HIF-1*α* by inhibing mTor	Oza et al. [51]
	Everolimus (RAD001)		Slomovitz et al. [52]

*Inhibitors of HIF-1 target genes*			
VEGF inhibitor	Bevacizumab	Monoclonal antibody against VEGF	Aghajanian et al. [53]
CAIX inhibitor	Rencarex (WX G250)	Monoclonal antibody against CAIX	—
